# Differential co-expression network centrality and machine learning feature selection for identifying susceptibility hubs in networks with scale-free structure

**DOI:** 10.1186/s13040-015-0040-x

**Published:** 2015-02-03

**Authors:** Caleb A Lareau, Bill C White, Ann L Oberg, Brett A McKinney

**Affiliations:** 1 Tandy School of Computer Science, Department of Mathematics, University of Tulsa, Tulsa, OK USA; 2 Division of Biomedical Statistics and Informatics, Department of Health Sciences Research, Mayo Clinic, Rochester, MN USA; 3 Mayo Clinic Vaccine Research Group, Mayo Clinic, Rochester, MN USA; 4 Laureate Institute for Brain Research, Tulsa, OK USA

## Abstract

**Background:**

Biological insights into group differences, such as disease status, have been achieved through differential co-expression analysis of microarray data. Additional understanding of group differences may be achieved by integrating the connectivity structure of the differential co-expression network and per-gene differential expression between phenotypic groups. Such a global differential co-expression network strategy may increase sensitivity to detect gene-gene interactions (or expression epistasis) that may act as candidates for rewiring susceptibility co-expression networks.

**Methods:**

We test two methods for inferring Genetic Association Interaction Networks (GAIN) incorporating both differential co-expression effects and differential expression effects: a generalized linear model (GLM) regression method with interaction effects (reGAIN) and a Fisher test method for correlation differences (dcGAIN). We rank the importance of each gene with complete interaction network centrality (CINC), which integrates each gene’s differential co-expression effects in the GAIN model along with each gene’s individual differential expression measure. We compare these methods with statistical learning methods Relief-F, Random Forests and Lasso. We also develop a mixture model and permutation approach for determining significant importance score thresholds for network centralities, Relief-F and Random Forest. We introduce a novel simulation strategy that generates microarray case–control data with embedded differential co-expression networks and underlying correlation structure based on scale-free or Erdos-Renyi (ER) random networks.

**Results:**

Using the network simulation strategy, we find that Relief-F and reGAIN provide the best balance between detecting interactions and main effects, plus reGAIN has the ability to adjust for covariates and model quantitative traits. The dcGAIN approach performs best at finding differential co-expression effects by design but worst for main effects, and it does not adjust for covariates and is limited to dichotomous outcomes. When the underlying network is scale free instead of ER, all interaction network methods have greater power to find differential co-expression effects. We apply these methods to a public microarray study of the differential immune response to influenza vaccine, and we identify effects that suggest a role in influenza vaccine immune response for genes from the PI3K family, which includes genes with known immunodeficiency function, and KLRG1, which is a known marker of senescence.

**Electronic supplementary material:**

The online version of this article (doi:10.1186/s13040-015-0040-x) contains supplementary material, which is available to authorized users.

## Background

In co-expression analysis, the correlation between pairs of genes is typically combined into a network model of the correlation structure, which facilitates secondary network analysis such as community structure or centrality [1]. However, the correlation between pairs of genes in a co-expression network typically is assumed to be uniform across all samples (*e.g.*, tissue types, treatment conditions, disease status, etc.). Yet it is often inter-group differences in correlated data that are of biological or clinical interest. For example, a gene co-expression network in microarray data for chronic lymphocytic leukemia using known biomarkers was able to predict treatment outcomes in an independent sample [2]. A differential co-expression network approach that leverages the genetic network information may yield novel biomarkers and improved prediction.

Differential expression methods compute the mean difference between groups for each gene but typically do not incorporate conditional variation from other genes in the data that may help explain the between-group variation. Differential co-expression computes the mean pairwise *correlation difference* between groups [3]. While the change in a gene’s expression may influence the phenotype in isolation or have only a single pairwise interaction with another gene, it is more likely that changes in a gene’s expression will have a cascading effect with the emergence of multiple differential co-expression effects due to the underlying biological network structure. In the current study, we combine differential expression and differential co-expression effects into a single network model and determine the importance of genes based on a network centrality score that models additional phenotypic variation from gene expression data.

The ability to detect susceptibly hubs in differential gene expression networks depends on the properties of the underlying biological network. For example, scale-free networks exhibit a power-law distribution and are characterized by having a few hubs and many nodes with low degree [4]. It is known that the targeted mutation of hubs (central proteins) in yeast protein-protein interaction networks is more likely to be lethal than the mutation of low degree (non-central) proteins, which is referred to as the centrality-lethality rule [5]. A variety of biological networks have displayed evidence of scale-free behavior, such as metabolic networks [6], protein–protein interaction networks [7] and transcriptional networks [8].

For a scale-free biological network the probability of a random mutation occurring to a hub is small relative to non-hubs; thus, hubs may be probabilistically insulated by the presence of many non-central nodes. Despite this protected status of hubs, there is a potential for hubs to show a differential co-expression effect without themselves being mutated. The potential for this side-effect can be understood by noting that random mutations are more likely to occur to non-hubs, but a mutated non-hub has a high probability of being connected to a hub and, hence, this hub may show differential co-expression despite not being mutated.

In previous work we developed a variation of PageRank centrality to find susceptibility hubs for epistasis network analysis of rare and common variant data [9-11]. We utilized this centrality with our epistasis network inference method called regression-based Genetic Association Interaction Network (reGAIN) that combines main effects and epistatic effects into a network model of a given phenotype. Epistasis is the deviation from the additive effect of DNA variants, but a similar effect can be observed at the expression level, where the phenotypic effect of one gene is modified depending on the expression of another gene [12,13]. Differential co-expression represents an example of this more general “expression-epistasis” effect.

Testing on simulated data is important for validating the proposed differential network methods. However, there is a lack of methods to simulate artificial gene expression data with differential co-expression or expression-epistasis effects while also including a realistic underlying network structure. Thus, we introduce a network-based simulation algorithm for constructing artificial gene expression data sets to assess the ability of statistical methods to identify significant hubs of differential co-expression. The underlying networks are designed to have degree distributions that may be either scale-free or Erdos-Renyi (ER) random.

This network simulation strategy, which uses specific degree distributions and random disruptions to the correlation within the case group, allows us to address the effect of the degree distribution on the ability to detect differential co-expression effects, network transitivity and other network effects. We assess the true and false positive rates under a variety of simulation conditions. We compare the ability to identify genes involved in differential co-expression for different edge inference approaches, including Fisher transformed z-test for differential correlation (dcGAIN) with a *t*-test on the diagonal and the generalized linear regression model (reGAIN).

Unlike statistical inference testing methods based on analytical null-hypothesis distributions, network centrality scores and Relief-F scores lack an analytical null distribution. Thus, we use permutation and mixture model density estimation to determine critical values of the centrality scores for null model rejection for genes in the network. The mixture model concept is similar to approaches for modeling microarray data p-values as a mixture of null and alternative densities [14,15]. We also use the mixture model and permutation methods to find statistical thresholds for machine learning comparison methods Relief-F [12,16] and Random Forest [17] importance scores. To understand the role of main effects in the network, we include Lasso as a comparison method [18]. In addition to realistic artificial data, we apply the methods to a seasonal influenza study with pre- and post-vaccination microarray and antibody response data [19].

## Methods

### dcGAIN and reGAIN for constructing the interaction network

Prior to centrality analysis for ranking genes, described below, we must construct a matrix that encodes the statistical interaction or differential co-expression between genes. There are multiple ways to calculate the statistical interaction between genes in a genetic association interaction network (GAIN). Here we describe two methods for constructing the matrix elements of the interaction network.

#### Regression GAIN (reGAIN) using the generalized linear model

To model differential co-expression between transcripts in reGAIN, we use the generalized linear model with a full interaction logistic regression model [20]:

1 $$ 1\mathrm{n}\left(\frac{ \Pr \left(D=1\left|{G}_i\right.{G}_j\right)}{ \Pr \left(D=0\left|{G}_i,{G}_j\right.\right)}\right)={b}_b+{b}_i{G}_i+{b}_j{G}_j+{b}_{ij}{G}_i{G}_j $$

The values of the outcome variable D are the case (D = 1) and control (D = 0) status. The predictor variables are the gene expression levels for genes *G*_*i*_ and *G*_*j*_. In the reGAIN, we use the standardized coefficient for the multiplicative interaction term, b_ij_, as the off-diagonal elements of the interaction matrix A in the gene centrality calculation (in Eq. 5). The diagonal elements of the reGAIN matrix are the regression coefficients for a single-gene model.

#### Differential co-expression GAIN (dcGAIN) *using the Fisher Z-test*

To model differential co-expression in dcGAIN, we use the Fisher Z-test [21] by the following steps. First the correlation is calculated between pairs of genes i and j for subjects within each phenotype group, where the groups again are specified by D = 1 (cases) and D = 0 (controls):

2 $$ {r_{ij}}^{(D)}=\frac{\operatorname{cov}\left({G}_i,{G}_j\right)}{\sigma_{G_i}{\sigma}_{G_j}} $$

The within-group correlation values are Fisher z-transformed:

3 $$ {Z_{ij}}^{(D)}=\frac{1}{2}1\mathrm{n}\left|\frac{1+{r_{ij}}^{(D)}}{1-{r_{ij}}^{(D)}}\right| $$

Finally the following test statistic is computed for the difference of the z-transformed correlation between groups D = 1 of size m_1_ and D = 0 of size m_0_ for genes i and j:

4 $$ {Z}_{ij}=\frac{\left|{Z}_{ij}^1-{Z}_{ij}^0\right|}{\sqrt{\frac{1}{m_1-3}+\frac{1}{m_0-3}}} $$

For the dcGAIN off-diagonal elements of A in Eq. (5), we use Z_ij_, the Fisher Z-test for inter-group difference in correlation between genes i and j. For the dcGAIN diagonal elements of A, we use a *t*-test for the individual genes.

### Interaction network centrality

To compute the importance of genes that show differential co-expression or statistical interaction effects at the expression level (expression epistasis), we use a generalization of a centrality approach that we developed for epistasis networks from GWAS data, called SNPrank [9-11]. Here we briefly discuss the relevant aspects of the method and modifications for gene expression interaction networks. This algorithm operates on a network, encoded as a weighted matrix, A, with diagonal terms that correspond to the main effect association of the gene on the phenotype and off-diagonal terms that correspond to the interaction effect on the phenotype or differential co-expression. The matrix elements of A are created either with dcGAIN or reGAIN, discussed above, and then we use the Complete Interaction Network Centrality (CINC) method to calculate each gene’s importance. “Complete” refers to the inclusion of main effects and interactions.

The CINC works by solving the following system of N equations for the vector R whose values correspond to the CINC centrality score for the corresponding gene or transcript i:

5 $$ {R}_i=\left\{\begin{array}{l}\frac{A_{ii}}{N\cdot Tr(A)}+\frac{1-\gamma }{N}+\gamma {\displaystyle \sum_{j\ne i}^N\frac{A_{ij}\cdot {R}_j}{k_j},\ }{k}_j\ne 0\\ {}\kern3.5em \frac{A_{ii}}{N\cdot \mathrm{T}\mathrm{r}(A)}+\frac{1}{N},\kern5em {k}_j=0\kern0.75em ,\end{array}\right. $$

where N is the number of genes and A is a weighted matrix of size NxN. For reGAIN, the diagonal elements of A correspond to main effect estimates from a single gene logistic model and off-diagonal elements correspond to statistical interactions b_ij_ in Eq. (1). For dcGAIN, off-diagonals are the Z_ij_, the Fisher Z-test for inter-group difference in correlation between genes i and j, and diagonal elements of A are t-tests for the individual genes. The Tr(A) is the trace of the A matrix, k_j_ is the jth element of the weighted degree vector of A (row sums of A), and *γ* is the so-called damping factor, which we usually assign the value 0.85 based on simulation studies of epistasis networks [11]. The 1/N terms in Eq. 5 give all genes a uniform baseline importance. One can see from this equation that the importance of gene i depends on its main effect (A_ii_) and the linear combination of importance scores of all of its connections (A_ij_*R_j_).

### Statistical thresholds for determining significance

A statistical distribution is not known for network centrality scores or Relief-F importance scores for calculation of statistical significance. Thus, we implement two approaches for setting statistical thresholds for significant gene associations from the centrality and importance scores, namely, a permutation approach and a mixture model approach.

#### Permutation algorithm

The permutation algorithm to determine significance is as follows:

- Compute observed data importance scores for all genes.
- Permute the data class labels mPerm times and accumulate an array of mPerm scores for each gene.
- Find the 95^th^ percentile score threshold for each gene’s permutation score array.
- Compare the observed score of each gene (unpermuted data) with its permutation threshold.
- Count the gene as a significant association if the gene’s score exceeds the threshold, else gene is non-significant

#### Mixture model algorithm

Due to the computationally intense nature of permutation, we also propose a two-mode Gaussian mixture model (GMM) clustering approach to the centrality, Relief-F and Random Forest scores to determine whether a gene’s score comes from the null density or the alternative density. We assume the density of scores comes from a linear combination of Gaussian distributions, which we find is a reasonable reflection of the scores. We use expectation maximization to estimate the parameters for the null and alternative densities. For each gene’s score, we compute the likelihood that the score belongs to the null density and the likelihood that the score belongs to the alternative density. The gene is classified as a significant (or non-significant) depending on whether the likelihood for belonging to the alternative is greater (or less) than the null likelihood.

### Network simulation strategy

**Figure 1:**
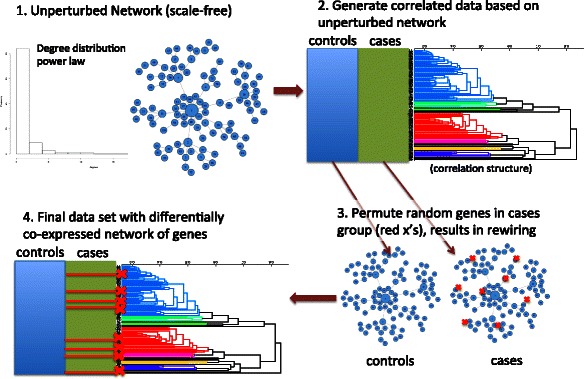
**The simulation of gene expression data with differential co-expression network effects begins with a gene network with given connectivity and degree distribution, such as scale-free (Step 1).** Initially the data set, with N genes and M subjects, has correlation structure that does not differ between groups (Step 2). A detailed algorithm for Step 2 is given in Figure 2. Briefly, the data set is initialized to a random Gaussian matrix, and then genes are changed to be proportional to others based on their connections in the adjacency matrix (Step 1). The strength of the correlation is regulated by a Gaussian (0, noise) variable, where smaller noise creates stronger correlation between genes. To create differentially co-expressed genes (Step 3), we arbitrarily split the M columns of data into two groups (cases and controls) and select random genes for permutation (red x’s) in the cases group. Note that this permutation is distinct from the permutation used to assess significance. This permutation keeps the simulated group means the same, so there are no main effects, but disrupts the wiring or correlation in the cases group between the target gene and the genes it was connected to in the adjacency matrix from Step 1. The co-expression in the healthy control group is left unchanged, resulting in a complex data set with an embedded differential co-expression network.

**Figure 2:**
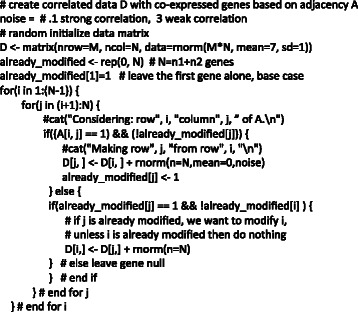
**Code for simulating gene expression data set, D, with N genes by M subjects with co-expression based on a scale-free or other degree distribution (additional details for step 2 in Figure****1****).** The input adjacency matrix A specifies the gene-gene correlation structure (from step 1 of Figure 1), and the variable “noise” determines the strength of the correlation. The data set is initially random Gaussian, and then a loop sets a gene’s expression proportional to another gene if they are connected according to the adjacency matrix and if the gene has not been already modified. In a subsequent step (step 3 in Figure 1), differential co-expression is added between cases and controls.

For this study, we develop a strategy for simulating case–control microarray data with differentially co-expressed genes and differentially expressed genes (outlined in Figures 1 and 2). The strategy builds a dataset with a baseline correlation network structure followed by “random attacks” of genes in the disease/cases group to disrupt co-expression of the attacked genes’ connections. Thus, differential co-expression emerges through the random disruption of the underlying correlation structure. The first step involves simulating an initial connectivity, encoded as an undirected adjacency matrix. This adjacency matrix is the wiring of the healthy control co-expression, and it is the starting point for rewiring the disease (cases) co-expression. We constrain the initial adjacency matrix to have one of two degree distributions: scale-free or ER. For the scale-free simulations, we use the preferential attachment algorithm [4]. While there is a great deal of evidence for scale-free networks in biology, it is not clear that this is always the case for co-expression networks. Thus, as an alternative we test network construction and centrality feature selection algorithms for simulated ER networks, which use a uniform probability to determine whether or not nodes are connected. An example of a scale-free network is shown in Figure 1 (step 1) with the corresponding power-law degree distribution.

For a given set of simulation parameters, we create 100 replicate data sets. The simulation parameters are the number of permuted genes that cause differential co-expression (n), the total number of genes (N), the sample size (M = cases + controls), the noise (standard deviation) in the correlation between genes, and fold change. We introduce two types of randomness for each replicate: noise in the theoretical correlation structure and the genes selected for permutation. For the total number of genes we use N = 100. Thus, in practice, we assume filtering is performed agnostic to outcome such as low value and low variance. We consider sample sizes (M = cases + controls) from the relatively small 20 (10 cases and 10 controls) to the more moderate M = 40 samples. The amount of Gaussian noise (standard deviation) added to correlated genes ranges from .05 (strong correlation) to 3 (weak correlation). We also generate simulations that contain differential expression (main effects) with fold change up to 2-fold. A standard deviation of the gene intensity measurements on the base-two logarithmic scale value of 0.7 is realistic for genes that are expressed at moderate to high levels [22].

#### Performance metrics

For simulated data with N total genes and n perturbed genes, we define the true positive rate (TPR) as the number of genes with centrality scores assigned to the high GMM mode that were also perturbed (true positives) divided by the total number of perturbed genes (m positives). Similarly we define the false positive rate (FPR) as the number of genes with centrality scores assigned to the high GMM mode that were unperturbed in the simulated data (false positives) divided by the total number of genes that were unperturbed (N-n). Performance evaluation works similarly for the permutation test approach for identifying significant centrality weights.

### Microarray data

We apply Relief-F, Lasso, dcGAIN and reGAIN network construction with CINC to a publicly available influenza vaccine dataset (GEO: GSE29619). We adjust for sex in the reGAIN models, which cannot be done easily with the other methods. In this study, 28 healthy adults were vaccinated during the 2008 influenza season with trivalent inactivated influenza vaccine (TIV) while measuring gene expression levels before and 7 days after vaccination [19]. Antibody titers against the influenza virus were recorded before and 28 days after vaccination. For our statistical analyses, we employed the day 7 versus baseline gene expression change as the predictors and high and low antibody titers at day 28 as the phenotype as defined in Ref. [19]. Similar to the goal of the original study, our application seeks to identify genetic effectors associated with differential immune response. However, while the previous study only analyzed the individual effects of gene expression on the antibody titer phenotype, our combined network approach accounts for both individual and interactive effects when prioritizing genetic effects.

## Results

### Simulation analysis

**Figure 3:**
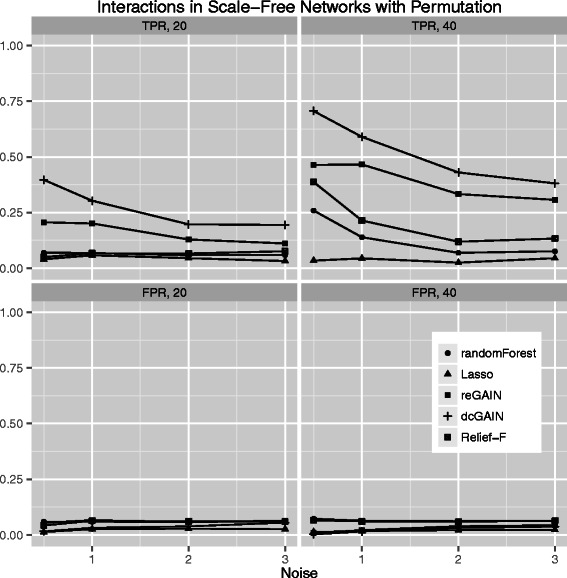
**Comparison of true positive and false positive rates to detect the 10% of genes involved in differential co-expression in Scale-Free networks in 100 replicate simulated data sets for increasing correlation noise (standard deviation) in the network.** True positive rates are on top panels and false positive rates are on bottom panels. Sample sizes are M = 20 (left panels) and M = 40 (right panels). We use permutation testing to determine the significance thresholds for Relief-F, Random Forest, reGAIN plus CINC and dcGAIN plus CINC.

**Figure 4:**
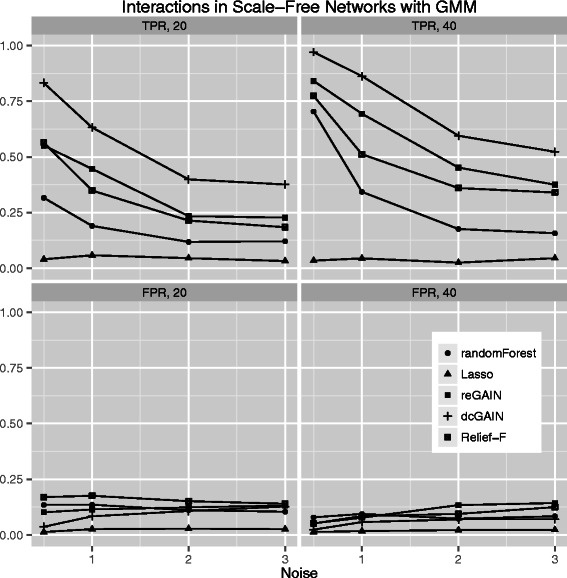
**Comparison of true positive and false positive rates to detect the 10% of genes involved in differential co-expression in scale-free networks in 100 replicate simulated data sets for increasing correlation noise (standard deviation) in the network.** True positive rates are on top panels and false positive rates are on bottom panels. Sample sizes are M = 20 (left panels) and M = 40 (right panels). We use Gaussian mixture modeling (GMM) to determine the significance thresholds for Relief-F, Random Forest, reGAIN plus CINC and dcGAIN plus CINC.

**Figure 5:**
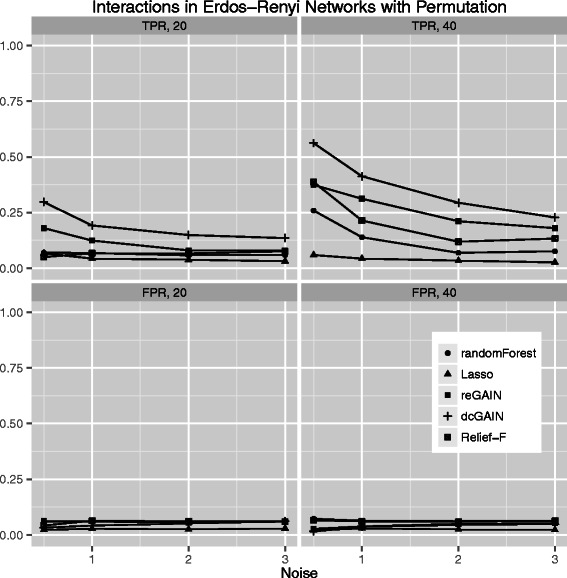
**Comparison of true positive and false positive rates to detect the 10% of genes involved in differential co-expression in Erdos-Renyi networks in 100 replicate simulated data sets for increasing correlation noise (standard deviation) in the network.** True positive rates are on top panels and false positive rates are on bottom panels. Sample sizes are M = 20 (left panels) and M = 40 (right panels). We determine the significance thresholds for Relief-F, Random Forest, reGAIN centrality and dcGAIN centrality with permutation testing.

**Figure 6:**
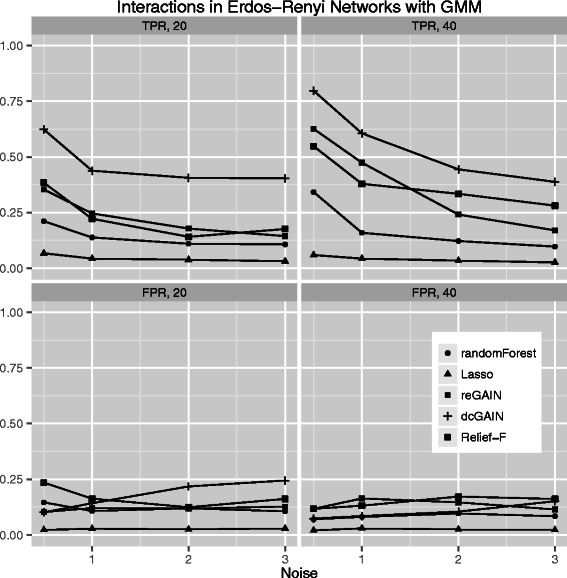
**Comparison of true positive and false positive rates to detect the 10% of genes involved in differential co-expression in Erdos-Renyi networks in 100 replicate simulated data sets for increasing correlation noise (standard deviation) in the network.** True positive rates are on top panels and false positive rates are on bottom panels. Sample sizes are M = 20 (left panels) and M = 40 (right panels). We determine the significance thresholds for Relief-F, Random Forest, reGAIN centrality and dcGAIN centrality with Gaussian mixture modeling (GMM).

The main goal of this study is to test the performance of methods to identify genes that are involved in changes in co-expression between groups. Thus, we compare the true positive rates (TPR) and false positive rates (FPR) for detecting the 10% of genes that were targeted in the simulated differential co-expression data. The models include varying amounts of network correlation noise (standard deviation) and either 20 or 40 samples (balanced cases and controls). We calculated the TPR and FPR for each method across 100 replicates for each model. For scale-free differential network simulations and the permutation method for assessing significance (Figure 3), we find the following TPR order (highest to lowest): dcGAIN + CINC, Relief-F, reGAIN + CINC, Random Forest and Lasso. The FPR for all methods are very low for all methods using permutation (Figure 3). When using GMM to determine significance (Figure 4), we find higher TPR for all methods with the same relative order as with permutation. However, permutation has the advantage of lower FPR compared with the GMM. For simulated ER networks, the methods have lower TPR than their analysis of scale-free differential co-expression networks but also slightly lower FPR (Figures 5 and 6). Lasso has very low TPR for all differential network simulations because the Lasso only includes main effect terms.

**Figure 7:**
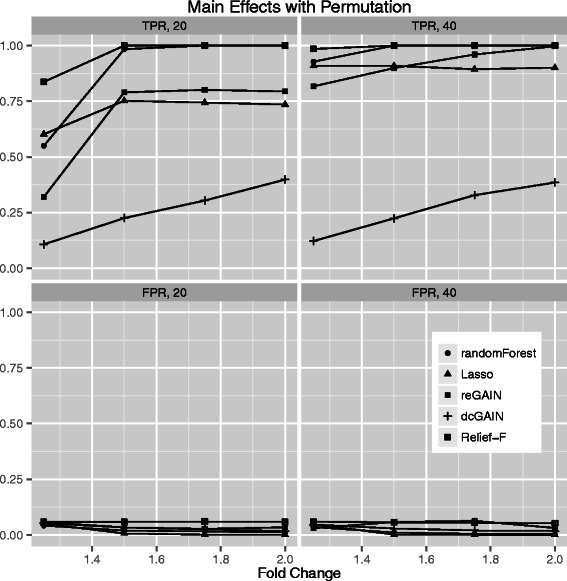
**Comparison of true positive and false positive rates to detect the 10% of main effect genes for different fold changes in 100 replicate simulated data sets.** In each plot, the fold change increases from left to right. True positive rates are on top panels and false positive rates are on bottom panels. Sample sizes are M = 20 (left panels) and M = 40 (right panels). We determine the statistical thresholds for Relief-F, Random Forest, reGAIN + CINC and dcGAIN + CINC centrality with permutation testing.

**Figure 8:**
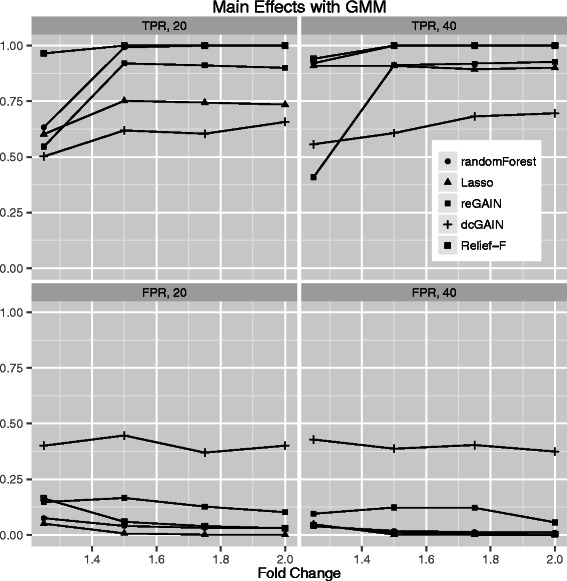
**Comparison of true positive and false positive rates to detect the 10% of main effect genes for different fold changes in 100 replicate simulated data sets.** In each plot, the fold change increases from left to right. True positive rates are on top panels and false positive rates are on bottom panels. Sample sizes are M = 20 (left panels) and M = 40 (right panels). We use Gaussian mixture modeling (GMM) to determine the statistical thresholds for Relief-F, Random Forest, reGAIN plus CINC and dcGAIN plus CINC.

In addition to differential co-expression effects, we expect many genes to show individual (main effect) differential expression between groups. Thus, we compare the TPR and FPR of the panel of methods to detect main effects. The simulations are similar to the differential co-expression simulations but instead of varying the correlation noise, we increase the effect size of the 10% of main effect genes up to 2-fold. For the permutation testing to determine significance (Figure 7), we find the order of TPR performance is Relief-F, Random Forest, reGAIN + CINC, Lasso, and dcGAIN + CINC. The main effect TPRs for all methods are similar with the exception of dcGAIN + CINC, which is distinctly lower than the others. Using GMM for significance in the main effect simulations (Figure 8), the TPRs are higher than the permutation testing approach (Figure 7) but follow the same trend. As is the case for the interaction simulations with GMM and permutation (Figures 3 and 4), the main effect simulations with GMM tend to have higher FPR (Figure 8) than permutation testing (Figure 7). While dcGAIN + CINC performs best for the interaction simulations, it performs worst for main effects. Relief-F shows the best overall performance at detecting both main effects and interaction effects. As with interaction simulations, these methods show higher TPR when using GMM, but permutation has the advantage of lower FPR. For computational expediency, we simulate only 100 genes with 10 target genes for each replicate because we create 100 replicate data sets for each simulation scenario and we use permutation testing in many cases (Figures 3, 5 and 7). However, we show for real data that all probes can be analyzed, though in practice filtering is recommended.

### Microarray analysis of differential immune response to influenza vaccine

We apply the feature selection methods reGAIN + CINC, dcGAIN + CINC, Relief-F and Lasso to a publicly available influenza vaccine dataset (GEO: GSE29619), results in Additional file 1: Table S1. For the interaction network based methods, we first filter the gene expression probes to the top 5,000 probes based on univariate regression p-values, though genes with no univariate effect could be implicated through only interactive effects. Since this filter is not aggressive, we do not cross validate this step even though it was not agnostic to outcome. After filtering the original probes, we apply dcGAIN and reGAIN to the 5,000 remaining transcripts. After each network was constructed, we apply the CINC centrality method to identify the most significant hubs and other effects in each network.

Significant genes for reGAIN + CINC and Relief-F show enrichment of PI3K-related pathways. Reactome pathways enriched in Relief-F and reGAIN include “Genes involved in Negative regulation of the PI3K/AKT network” (6.3e-4) and “Genes involved in PI3K cascade” (1.72e-3). The specific genes found include PIK3R5 and AKT3 – found by both reGAIN and Relief-F – and PTEN and FGF23, found by Relief-F only. The PIK3R5 gene was also selected by Lasso but not dcGAIN, indicating a consensus among methods for the main effect of this gene. This PI3K pathway signature is biologically relevant to differences in influenza vaccine immune response because loci in the PIK3CD gene are associated with an immunodeficiency syndrome that presents with recurrent respiratory infection, increased circulating transitional B cells, and impaired vaccine response. A recent study found a gain-of-function rare variant (nonsynonymous) in PIK3CD for the syndrome and increased levels of phosphorylated AKT protein from patient-derived lymphocytes [23].

**Figure 9:**
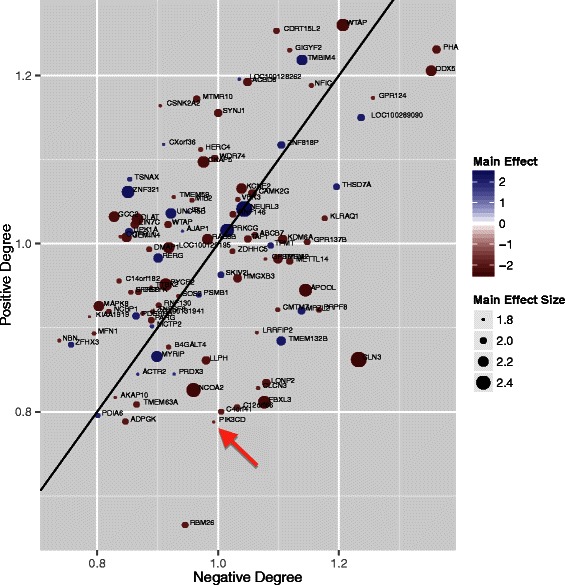
**Differential co-expression hub scatter plot from a microarray study of high and low immune responders to influenza vaccine.** The horizontal axis measures the magnitude of the sum of the negative interactions that each gene has with other genes, where a negative interaction increases the probability of being a low immune responder to the vaccine. The vertical axis measures the positive interactions of each gene. The diagonal line represents the null expectation of a gene having the same number of positive and negative interactions. The size of a plot point indicates the size of the main effect of the gene and the color indicates whether the gene has a positive or negative effect on immune response. The arrow highlights an important gene (PIK3CD) due to its having a strong negative hub effect (below the diagonal line) but very little main effect (small plot point radius).

Differential co-expression hub analysis (Figure 9) reveals that PIK3CD has a strong negative hub effect but very low main effect on immune response. This type of epistasis analysis tool is similar to that used for visualizing interaction effects from double mutant strains of yeast [24]. A negative interaction for the vaccine immune response outcome represents a joint effect that leads to decreased immune response to the vaccine. A negative *hub* is a gene whose sum of negative interactions outweighs its positive interactions; such genes fall below the null line (Figure 9). In addition to global interaction effects, the positive/negative hub plot also includes main effect information based on size and color of the plot symbol. For example, PIK3CD, which has been shown to affect immune response to vaccination, displays an important effect as a negative differential co-expression hub. However, PIK3CD has a negligible effect by univariate analysis of this microarray study, indicated by the small plot symbol for the gene.

Our simulation results show that dcGAIN was the highest performer at finding interactions but worst at finding main effects. This is corroborated in the data analysis in which the dcGAIN + CINC results have the smallest intersection of genes with Lasso (only 1 out of 17 genes). Whereas Relief-F find 13/17 and reGAIN + CINC find 5/17 main effect genes. Thus, many of the genes for differential immune response to influenza vaccine found by dcGAIN are likely due to substantial interaction effects.

For example, one of the dcGAIN hubs is the killer cell lectin-like receptor G1 (KLRG1), which has been used to define populations of senescent effector CD8 T cells in mice and humans [25]. Additionally, influenza virus-specific CD8 T cells showed a decrease in functionality corresponding to increases in KLRG1 [26]. Moreover, another interaction hub identified by the dcGAIN + CINC approach was the cyclin-dependent kinase 13 (CDK13) gene, which has been linked to increase viral production [27]. Taken together, these observations indicate that a differential correlation network structure has the power to uncover biological effectors that implicate both general and specific immune processes that other statistical methods do not uncovered. In addition to the PI3K pathway, the novel link to general viral regulator gene (CDK13) and a specific influenza T cell gene using transcriptomics (KLRG1) demonstrate the utility of our proposed framework.

## Discussion

Most gene expression analyses focus on identifying genes or sets of genes that show differential expression between phenotypes based on a univariate statistic. However, the co-expression between two genes may be conditional on the phenotypic or biological context. In other words, pairs of genes may be differentially co-expressed, whereby the wiring between two genes in a healthy or homeostatic network switches or is disrupted in a disease or perturbed network. Furthermore, influential gene hubs that discriminate between phenotype may be identified through the agglomeration of the univariate and pair-wise interactions in a condition specific gene network model. We used two methods for estimating the edges in these genetic association interaction networks (GAIN): Fisher z-test for differential correlation (dcGAIN) and a GLM regression model approach with gene-gene interaction terms (reGAIN). We applied our interaction network centrality algorithm (CINC, Eq. 5, a generalization of SNPrank for GWAS) to identify important susceptibility hubs and candidate genes for network rewiring. In addition, we compared network feature selection methods with Relief-F, Random Forests, and Lasso.

In order to assess the effects of correlation structure in a controlled way, we introduced a differential co-expression network simulation strategy that incorporated realistic network structures such as scale-free and ER, and we used random mutation to induce differential co-expression. As expected, Lasso was unable to detect the simulated differential co-expression effects because we did not include interactions in the Lasso. The other methods, which model conditional dependence between genes, tended to have greater power to detect susceptibility genes in the scale-free networks compared with ER. For reGAIN and dcGAIN, this increased power in scale-free networks is partly attributable to CINC centrality; centralities are sensitive to hub effects, and scale-free networks are characterized by hubs, whereas nodes in ER networks have uniform degree on average. Regardless of the underlying network degree distribution, dcGAIN had the highest power to detect the differential co-expression effects, followed by reGAIN and Relief-F – which had very similar performance – and finally Random Forest had the lowest power. We have shown previously that Random Forest is limited in its ability to find gene-gene interactions [28]; however, it performed reasonably well in these interaction simulations because we limited the number of simulated background genes.

Although our motivation for using network and machine learning approaches was to detect additional variation due to interaction effects, for completeness we also tested these approaches on main effect simulations. While dcGAIN performed best for differential co-expression interactions, it had the lowest power and highest false positive rate than the other methods for main effects. The test statistics for differential correlation on the off-diagonal tended to be larger than the test statistics for the main effect tests on the diagonal in these simulations, even in the absence of differential correlation. It may be that the larger number of off-diagonal differential correlation terms (n (n-1)/2 of them) masks the smaller number of main effect terms (n of them) in the CINC statistic. This discrepancy between dcGAIN interaction and main effect detection perhaps may be addressed by bringing dcGAIN into a regression framework. The main effects pose less difficulty for reGAIN, which uses regression coefficients for the diagonal and off diagonal. Relief-F performs best for main effect simulations, which, coupled with its relatively good performance on interactions, suggests Relief-F is a good all-purpose filter.

One of the challenges for model-free feature selection methods, like network centralities or Relief-F, is determining the statistical significance of feature scores. Thus, we introduced two methods to assess the statistical significance of the CINC centrality scores for dcGAIN and reGAIN and for Relief-F and Random Forest importance scores: a mixture model approach and permutation testing. The mixture model approach tends to give greater power and greater computational speed, but at the expense of more false positives than the permutation approach. On a related note, we do not use permutation when calculating the GLM interaction models of reGAIN, but instead we use the usual p-values and standardized beta coefficients. It has been shown for genetic data that permutation must be implemented carefully to handle the simultaneous interaction and main effect null hypotheses [29].

The Fisher z-test used in dcGAIN is designed to find correlation differences between groups, which made it better than reGAIN at finding the differential co-expression effects constructed in our simulations. However, the GLM framework used by reGAIN can be used for quantitative traits and any phenotype that can be modeled with an exponential family distribution, including time-to-event phenotypes. Further, the GLM framework can adjust for covariates, like sex, which is known to affect immune response. Thus, reGAIN + CINC provides more modeling flexibility plus it balances interaction and main effects better than dcGAIN.

Our application of these interaction network methods to a microarray study of the differential immune response to influenza vaccine found novel markers that are missed by main effect analysis. From our network centrality and machine learning analysis, we identified PI3K related genes, which have been previously demonstrated to be effectors in human immune responses [23] but would be missed by a univariate analysis of the influenza vaccine microarray study. Similarly, we found a differential co-expression network effect for the KLRG1 gene, which plays a role in immunosenescence in influenza virus specific CD8 T cells [26], and the CDK13 gene, which is associated with viral production [27], but a conventional differential expression analysis does not identify these genes as an effector. Ultimately, our findings implicate novel effectors in viral activity and validate previously identified influenza effects through transcriptomics analyses. The identification of these biomarkers combined with our simulated results demonstrate the advantages of using machine learning and differential co-expression network centrality to augment univariate approaches to identify functional effectors in microarray data.

## Additional file

Additional file 1: Table S1. Significant genes for high versus low HAI in baseline versus day-7 gene expression following TIV 2008 influenza vaccination (GSE29619).
